# Draft Genome Sequence of Chemolithoautotrophic Acetogenic Butanol-Producing Eubacterium limosum ATCC 8486

**DOI:** 10.1128/genomeA.01564-14

**Published:** 2015-02-12

**Authors:** Yoseb Song, Byung-Kwan Cho

**Affiliations:** a Department of Biological Sciences and KAIST Institute for the BioCentury, Korea Advanced Institute of Science and Technology, Daejeon, Republic of Korea; b Intelligent Synthetic Biology Center, Daejeon, Republic of Korea

## Abstract

Eubacterium limosum ATCC 8486 is an anaerobic chemolithoautotrophic acetogenic bacterium that converts and transforms syngas and isoflavonoids to butanol and phytoestrogens, respectively. Here, we report the draft genome sequence of the E. limosum ATCC 8486 (4.37 Mb) strain and its annotation information, including syngas fermentation and denitrification metabolic pathways.

## GENOME ANNOUNCEMENT

Eubacterium limosum ATCC 8486 is an obligate anaerobic and Gram-positive bacterium, which is capable of converting C_1_ compounds to complex carbohydrates or long-chain fatty acids. It is a major isolate of human intestinal contents and a type strain for the E. limosum species (1). The bacterium utilizes CO, CO_2_, and H_2_ as precursors (2) and produces butyrate, caproate, and acetate via microbial metabolism (3), known as syngas fermentation. Compared to traditional thermochemical processes like Fischer–Tropsch synthesis, syngas fermentation of acetogenic bacteria produces industrial commodities with higher catalyst specificity and lower energy costs (4). In particular, E. limosum is involved in the biotransformation of the isoflavonoids biochanin A, formononetic, and glycitein to the estrogenic metabolites genistein, daidzein, and 6,7,4′-trihydroxyisoflavone, respectively (5), for the anticancer, antioxidant, anti-inflammatory, cardioprotective, and enzyme inhibitory effects. However, the lack of genetic information on E. limosum hinders strain engineering and limits the understanding of its interaction with human and colonic bacteria. In this study, we obtained and analyzed the draft genome sequence and metabolic pathway of E. limosum in order to elucidate its physiological and metabolic properties.

E. limosum was cultivated under anaerobic conditions in basal media supplemented with 10% fructose (6). The isolation and fragmentation of genomic DNA was carried out using the PowerSoil DNA Isolation kit (Mo Bio Laboratories, Carlsbad, CA) and Covaris S220 (Covaris, Inc., Woburn, MA). The Illumina paired-end library was constructed from the genomic DNA, and sequenced using a TruSeq kit (Illumina, Inc., San Diego, CA) on the MiSeq v2 platform, with a 2 × 150-cycle paired-end read recipe. The obtained reads were trimmed on the CLC Genomics Workbench (CLC bio, Aarhus, Denmark) using default parameters. With an average read length of 150.35 bp, 582,633,852 bases were collected from 38,752,997 reads. The retrieved reads were then assembled using the CLC Genomic Workbench (minimum contig length, 402; automatic bubble size, yes; word size, 51; perform scaffolding, yes). rRNA and tRNA genes were predicted using the RNAmmer 1.2 (7) and tRNAscan-SE 1.31 (8), respectively. Annotation was performed with the Rapid Annotation using Subsystems Technology server (9).

The draft genome sequence of E. limosum ATCC 8486 is 4,370,113 bases, comprising 47.2% G+C content, 4,309 predicted open reading frames, 51 tRNA genes, and 11 rRNA genes, which are similar to those of E. limosum KIST 612 strain (4,316,707 bases; 47.5% G+C content) (10). However, the two strains displayed different carbohydrate metabolic pathways. For example, E. limosum ATCC 8486 demonstrated NADH-dependent butanol dehydrogenase (EC 1.1.1)-mediated conversion of acetyl-CoA to butanoyl-CoA. It also expressed enzymes affecting the denitrification pathway for energy generation such as nitrate reductase, nitrite reductase, nitric oxide reductase, and nitrous oxide reductase (11). With these genetic contents, the draft genome sequence of E. limosum would therefore assist in elucidating its interactions with human colonic bacteria.

### **Nucleotide sequence accession numbers**.

The draft genome sequence of E. limosum ATCC 8486 has been deposited in the DDBJ/EMBL/GenBank database under the accession no. JWIS00000000. The version described in this paper is the first version, JWIS01000000.
